# A Double Case: Socket Shield and Pontic Shield Techniques on Aesthetic Zone

**DOI:** 10.1155/2020/8891772

**Published:** 2020-10-29

**Authors:** Carlos Polis-Yanes, Carla Cadenas-Sebastián, Claudia Oliver-Puigdomenech, Raul Ayuso-Montero, Antoni Marí-Roig, José López-López

**Affiliations:** ^1^School of Dentistry, University of Barcelona, University Campus of Bellvitge, Barcelona, Spain; ^2^School of Dentistry, University of Barcelona, Oral Health and Masticatory System Group (Bellvitge Biomedical Research Institute) IDIBELL, University of Barcelona, University Campus of Bellvitge, L'Hospitalet de Llobregat, Barcelona, Spain; ^3^Department of Maxillofacial Surgery, University Hospital of Bellvitge, Catalonia, Spain; ^4^Oral Health and Masticatory System Group (Bellvitge Biomedical Research Institute) IDIBELL, University of Barcelona, L'Hospitalet de Llobregat, Barcelona, Spain; ^5^Department of Odontostomatology, Faculty of Medicine and Health Sciences (Dentistry), Odontological Hospital University of Barcelona-University of Barcelona/Oral Health and Masticatory System Group (Bellvitge Biomedical Research Institute) IDIBELL, University of Barcelona, L'Hospitalet de Llobregat, Barcelona, Spain

## Abstract

When a tooth is extracted, a bone remodeling of the alveolar process occurs irretrievably. Various techniques have emerged over time to maintain the thickness of the bone crest in fixed prosthetics on teeth and implants. The socket shield and pontic shield techniques are aimed at minimizing buccal bone remodeling, especially in the aesthetic area. We present a case of an aesthetic sector rehabilitated with partial fixed denture using the socket shield and pontic shield techniques.

## 1. Introduction

When a tooth is extracted, the healing of the socket inevitably involves reshaping of the periodontal alveolar process with horizontal and vertical bone loss. Postextraction immediate implant placement has been proven to be a reliable and safe process, with an osseointegration success rate similar to that of conventional implants, but it does not prevent bone remodeling after extraction [1]. It has also been suggested that various regenerative treatments, such as soft or hard tissue grafts, help to compensate this alveolar process reduction [1, 2].

Among bone grafts that have been studied for immediate implantology, we can find allogenic, xenogenic, and alloplastic grafts. All of them are successful, and they have minimal differences in reabsorption [1].

The socket shield technique (SST) was published for the first time in 2010 as a feasible technique to avoid buccal bone reshaping after tooth extraction, in combination with immediate implant placement. A thin buccal and interproximal layer or fragment of the tooth needs to be maintained in order to avoid buccal bone resorption [3].

## 2. Objectives

The purpose of this article is to present a clinical case of a patient who underwent immediate implant placement with the SS technique in the superior aesthetic area.

## 3. Case Report

**Figure 1:**
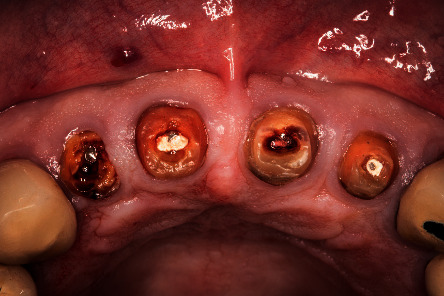
Image of the roots before the treatment.

**Figure 2:**
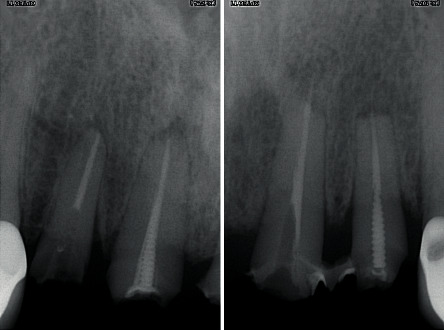
Periapical X-rays of the 4 incisive roots.

A 50-year-old man was referred to us having a decemented bridge from 1.2 to 2.2, with inadequate ferrule and metal posts in each root (1.1 and 2.2), both fractured (Figures 1 and 2). Given this miserable condition of the roots and their poor prognosis, rehabilitation of the area with fixed prostheses supported by two implants was proposed.

**Figure 3:**
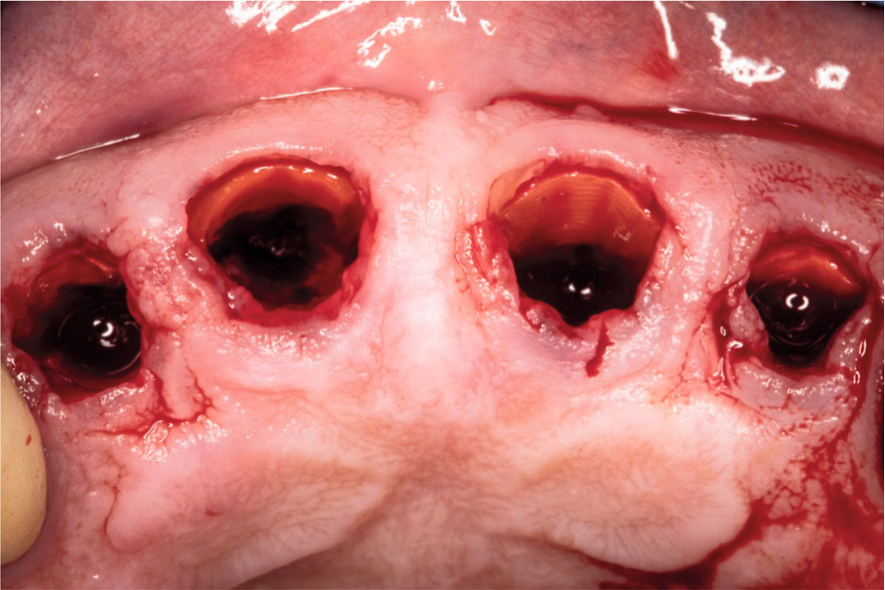
Intraoperative image of the root shield's preparation after extraction.

**Figure 4:**
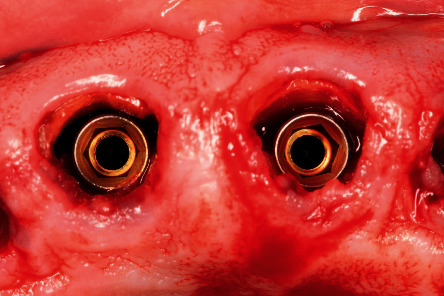
Intraoperative image of the implants placed, not being in contact with the buccal surface of the roots.

**Figure 5:**
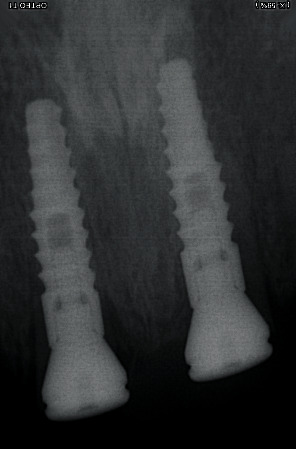
Postoperative periapical X-ray, taken immediately after implant placement.

The socket shield technique was performed on central incisors and pontic shield on lateral incisors (Figure 3) in order to minimize resorption and reshaping of the bone crest in implants and pontics. Haemostatic collagen sponges were placed both in 1.2 and in 2.2, and immediate implant placement was performed in 1.1 and 2.1 (Figures 4 and 5). In order to keep anterior guidance, lateral incisors were planned in cantilever, contactless during disclusion movements.

**Figure 6:**
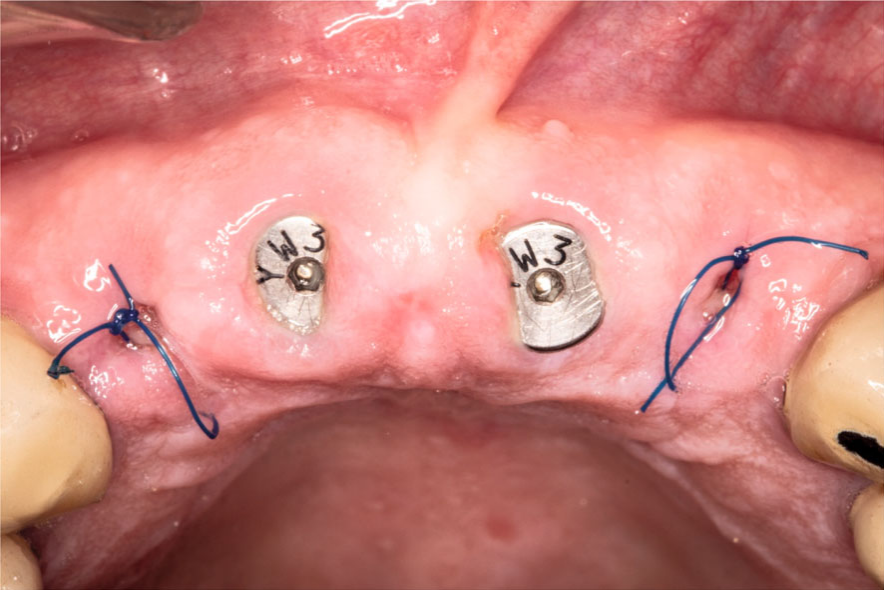
One-week follow-up.

Two Biohorizons Tapered Internal 3.8 × 12 mm implants with 3.5 mm diameter internal hexagonal connection (Biohorizons®, Spain) with an insertion torque of 25 N/cm were placed, as well as the healing abutments, because the patient did not request provisional teeth. After one week, healing was proper (Figure 6).

**Figure 7:**
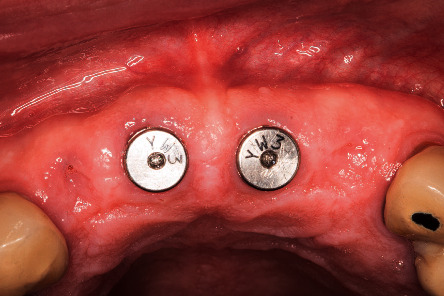
Three-month follow-up, after implant placement.

**Figure 8:**
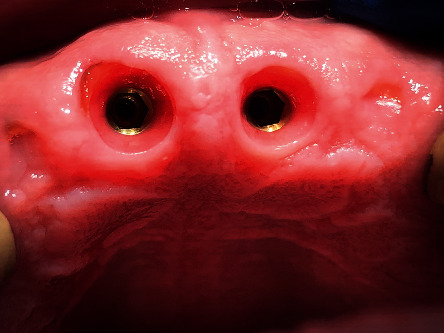
Shield exposure on 1.1 implant.

**Figure 9:**
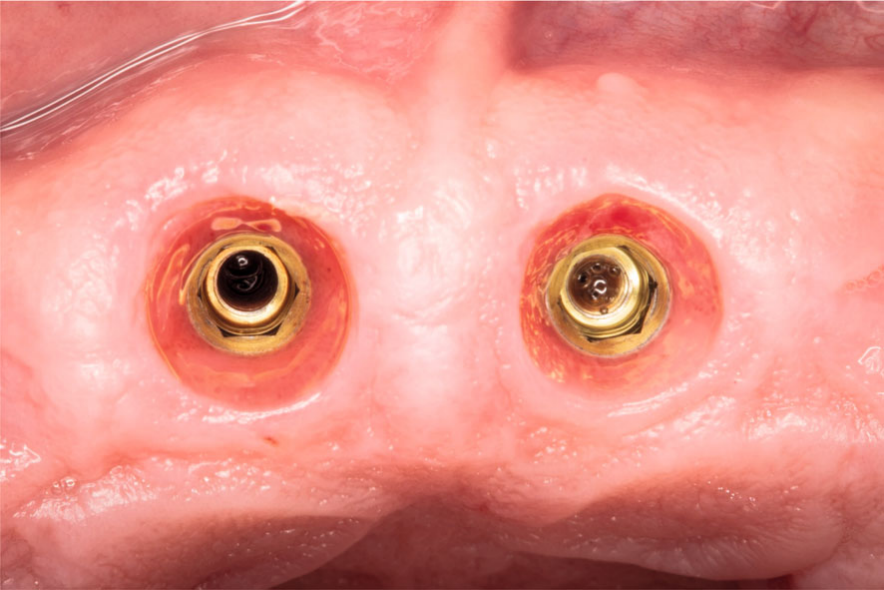
One-month follow-up, after high-speed handpiece reshaping of the shield exposure on 2.1.

**Figure 10:**
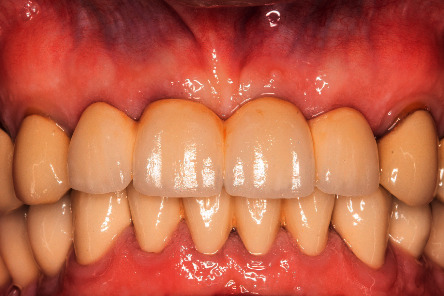
Final bridge delivery.

**Figure 11:**
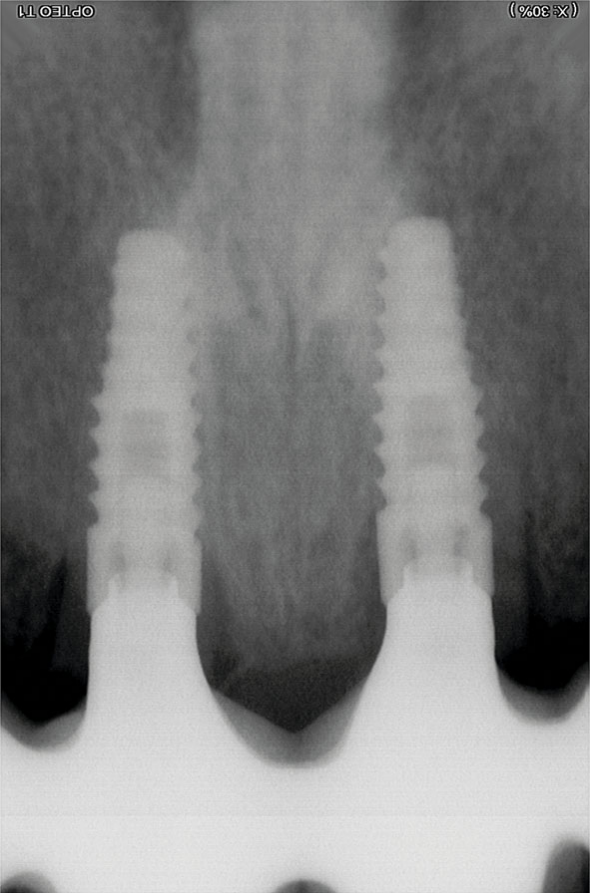
Control X-ray after bridge placing.

**Figure 12:**
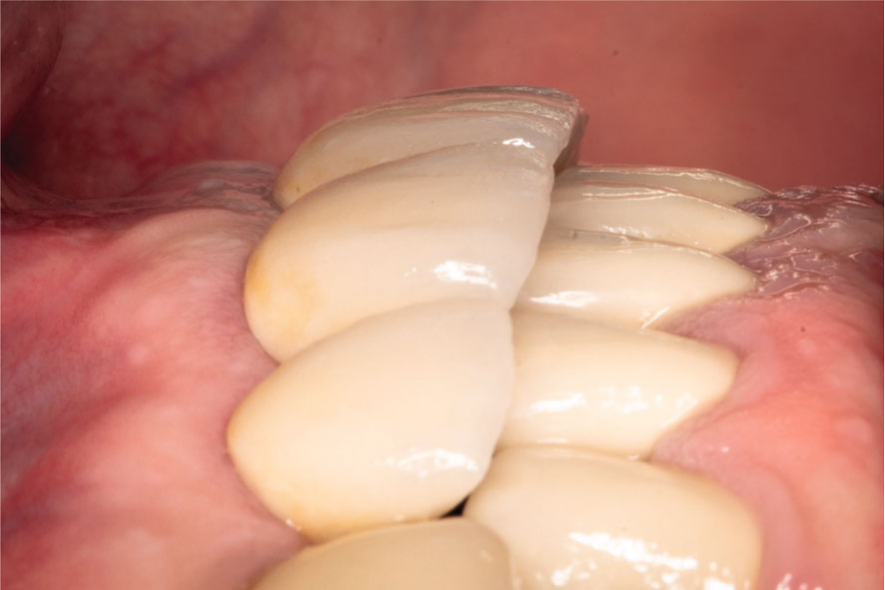
Clinical lateral vision. A good maintenance of the buccal thickness is seen.

Three months after, the osseointegration of the implants was corrected (Figure 7), but teeth 1.1 had a superficial exposure of the shield (Figure 8), which was reshaped with the high-speed handpiece using a diamond bur, without needing anesthesia. After one more month, the clinical appearance was corrected (Figure 9) and a partial fixed denture was screwed at 30 N/cm to the implant (Figures 10 and 11). Good maintenance of buccal volume and natural appearance is seen (Figure 12).

These partial extraction techniques have allowed us to maintain the volume of the buccal crest without the need to fill the gap with bone grafts and give the gingiva a natural appearance.

The authors want to emphasize that the value of this study is limited, since it is a case report of a single patient.

## 4. Discussion

Already in 2009, Davarpana and Szmukler-Moncler [4] presented a limited series of 5 cases with a follow-up between 12 and 42 months, in which the implants were drilled and placed through the roots of ankylosed teeth, leaving the fragments that remained stable in the socket. They found no complications derived from tooth remains in contact with the implant, and the cases were successful throughout the follow-up. They present the technique as an alternative to tooth extraction when it can cause morbidity.

Several studies have been published regarding the socket shield technique since its first reference by Hürzeler et al. [3]. Thus, Kan and Rungcharassaeng [5] and later Cherel and Etienne [6] used the SST in order to preserve the interdental papilla in between implants, in cases where adjacent teeth need to be restored.

Siormpas et al. [7] conducted a study on 46 patients with single implants, performing the “root-membrane” technique, similar to the TSS, and leaving the vestibular fragment at least 1 millimeter above the bone level and in contact with the implant surface. They obtained 100% success in a follow-up between 24 and 60 months and correct maintenance of the buccal bone volume. They concluded that it is a predictable technique for the placement of implants in aesthetic areas in healthy adults.

Gluckman et al. [8] described the pontic shield technique in which they performed a treatment similar to SST but, in these cases, filling the socket with xenogeneic graft in the pontics. Among the presented series, 13 cases were successful and one case required reshaping and advancement flap due to shield exposure. They conclude their study, describing the technique as a feasible option in bone preservation in pontics and assuming the need for more studies in this regard.

Afterwards, the same authors (in 2016 [9] and 2017 [10]) carried out two consecutive studies. The first study defined partial extraction techniques in three groups: (i) root submergence when the entire root volume is kept under the gum to preserve the volume, being very important the fact that the teeth do not have apical pathology; (ii) socket shield as a preparation of the root's buccal surface and 1 mm above the bony crest in anterior teeth and with simultaneous placement of immediate implants in the palatal surface of the socket, having eliminated any possible apical pathology before; (iii) pontic shield, which means preparing the socket shield exactly the same as SST but recommends filling the socket with osteoconductive material. They also advise soft tissue sealing and a three-month follow-up before pressing with ovoid pontics.  Table 1 shows the indications for the different partial extraction techniques based on this study and review. The study finishes by concluding that partial extraction techniques should be considered by clinicians as a conservative strategy to maintain bone crest in oral rehabilitation [9].

In his second work, he focuses on the technical aspects and the management of complications. The necessary materials for partial extraction techniques described in this article are presented in Table 2. They conclude by defining partial extraction techniques as a very promising set for the future management of bone ridges after the removal of nonpreservable teeth [10].

In 2017, Gharpure & Bhatavadekar wrote in a systematic review reporting limited scientific support regarding TSS, with evidence of less osseointegration, some biological complications and short follow-up cases. They suggest the need of more studies to obtain scientific support [11]. More recently, Han et al. [12] presented a modified TSS with a series of 40 implants maintaining the buccal shield at bone crest level and with an approximate thickness of 1.5 mm, without biological complications and with a 100% success rate. They conclude that modified TSS appears to be a successful procedure combined with immediate implants because the root surface does not interfere with osseointegration and is beneficial for the aesthetics and maintenance of bone.

Bramanti et al. [13] reported a sample of forty patients who received an immediate single implant in the esthetic zone with SST versus immediate implants with the conventional technique and filling the gap with bone graft. No clinical complications were recorded at 3, 6, and 36 months from implant placement. A lower rate of crestal bone resorption was recorded in the TSS group in all checks over time.

Based on the published studies and the case we present, we can confirm that complications arise more easily when the tooth fragment remains above the bone crest. The filling of the socket does not seem necessary when performing this technique since the blood clot achieves a regeneration of the socket and the shield prevents buccal reshaping.

## 5. Conclusion

The conclusion that we can draw from this case report and the subsequent review of the literature is that partial extraction techniques, such as the socket shield and the pontic shield, are procedures that should be considered in oral rehabilitation in selected cases.

This kind of intervention is dependent on the surgical technique of the operator, and its reproducibility must be assessed.

## Figures and Tables

**Table 1:** Partial extraction therapies (PET) and their indications, adapted from Siormpas et al. [7].

PET	Clinical situation(s) indicated
Root submergence	(i) Unrestorable tooth crown or tooth indicated for extraction absence of apical pathology (ii) Healthy amputated pulp or endodontic therapy completed intention to preserve the alveolar ridge (ii) Planned removable full or partial prosthesis (iv) Planned pontic site beneath fixed prosthesis (v) Cantilever pontic site as an alternative to two adjacent implants (vi) Actively growing young patient planned for implant treatment later (vii) Ridge preservation in conjunction with other PET

Socket shield	(i) Unrestorable tooth crown or tooth indicated for extraction tooth root with or without apical pathology (ii) Intention to preserve the alveolar ridge, specifically to prevent buccopalatal collapse (iii) Immediate implant placement (iv) Ridge preservation in conjunction with other PET

Pontic shield	(i) Unrestorable tooth crown or tooth indicated for extraction tooth root with or without apical pathology (ii) Intention to preserve the alveolar ridge (iii) Planned pontic site(s) beneath fixed prosthesis (iv) Cantilever pontic site as an alternative to two adjacent implants (v) Ridge preservation in conjunction with other PET

Proximal socket shield	(i) Unrestorable tooth crown or tooth indicated for extraction tooth root with or without apical pathology (ii) Intention to preserve interdental papillae (iii) Planned immediate implant placement sites of two or more adjacent implants (iv) Papillae preservation in conjunction with other PET

**Table 2:** Instruments and materials required for PET [8].

Socket shield
(1) Long shank root resection bur (2) Extralarge round diamond head bur (to reduce the inner aspect of shield into concavity) (3) End-cutting diamond head bur (to reduce coronal aspect of shield) 4. Gingival protector (4) Irrigated surgical motor (5) Contra-angled surgical fast handpiece (6) Microperiotomes (7) Microforceps

Pontic shield As for socket-shield, plus:
(1) Socket grafting instruments: plugger, particulate graft spoon, crucible (2) SM 69 blade (or other suitable microblade, mandatory for split thickness dissection of facial and palatal pouches to tuck CTG into) 3. 6/0 nylon sutures

Root submergence
(1) Irrigated surgical motor (2) Contra-angled surgical fast handpiece (3) Extralarge round diamond head bur (for reducing coronal aspect root into concavity) (4) SM 69 blade (or other suitable microblade, mandatory for split thickness dissection of facial and palatal pouches to tuck CTG into) (5) 6/0 nylon sutures
